# Advancing neuroblastoma care: future horizons after approval of eflornithine by FDA

**DOI:** 10.1097/JS9.0000000000001182

**Published:** 2024-02-13

**Authors:** Abubakar Nazir, Awais Nazir, Kamal Kandel

**Affiliations:** a Oli Health Magazine Organization, Research and Education, Kigali, Rwanda; b Department of Medicine, King Edward Medical University, Lahore, Pakistan; c Kathmandu University, Dhulikhel, Nepal

Neuroblastoma stands as the most prevalent cancer in children under 1-year-old and the primary solid tumor outside the central nervous system across all age ranges. The condition exhibits varied clinical behaviors, categorized into low, medium, and high-risk groups. Approximately half of neuroblastoma cases fall into the high-risk category, determined by factors like age, disease extent, histology, and cytogenetic features. While historically associated with poor outcomes, modern multimodal therapy has improved survival rates, with around 50% of children expected to survive. Current high-risk neuroblastoma (HRNB) treatment involves intensive chemotherapy, radiation therapy, autologous stem cell transplant (ASCT), and immunotherapy^1^. It comprises 15% of deaths related to pediatric cancer, with around 650 cases annually in the United States, equivalent to 10.2 per million children (65 per million infants). Despite a slight 0.4% change over time, there has been an overall improvement in 5-year mortality from 1975 to 2005, though subgroup-specific mortality presents a varied scenario^2^. The high-risk category experiences the most unfavorable outcome, displaying extensive metastatic spread to the bone marrow, bones, lungs, and liver. They undergo initial chemotherapy to decrease tumor size at both the primary and secondary sites, followed by extensive surgical removal, myeloablative chemotherapy, and stem-cell transplantation. Subsequently, a combination of maintenance chemotherapy and immunotherapy, specifically the monoclonal antibody dinutuximab targeting GD2 on neuroblastoma cells, is employed. This immunotherapy contributes to an enhanced 2-year event-free survival (EFS), increasing it from 46 to 66% in HRNB patients^3^.

On 13 December 2023, the FDA (United States Food and Drug Administration) granted approval for eflornithine (Iwilfin, USWM, LLC) to diminish relapse risk in adult and pediatric patients with HRNB who showed at least partial response to previous multiagent, multimodality therapy, including anti-GD2 immunotherapy. This marks the first FDA approval for a treatment aiming to reduce relapse risk in pediatric patients with HRNB^4^. Efficacy was assessed in an externally controlled trial comparing outcomes from study 3b (investigational arm) and study ANBL0032 (clinical trial-derived external control arm). Study 3b (NCT02395666) was a multicenter, open-label, non-randomized trial with two cohorts. In stratum 1, 105 eligible HRNB patients received eflornithine orally, twice daily, based on body surface area until progression, unacceptable toxicity, or up to 2 years. The trial aimed to compare outcomes to the historical benchmark EFS rate from study ANBL0032^4^. The external control arm included 1241 patients on the experimental arm of study ANBL0032, comparing various treatments to cis-retinoic acid alone in pediatric HRNB patients. Patients meeting comparative analysis criteria were matched (1:3) using propensity scores. The primary analysis included 90 Iwilfin-treated patients and 270 control patients from study ANBL0032.

The primary efficacy outcome was EFS, including disease progression, relapse, secondary cancer, or death. The protocol-specified primary analysis showed an EFS hazard ratio (HR) of 0.48 (95% CI: 0.27–0.85) and an overall survival (OS) HR of 0.32 (95% CI: 0.15–0.70). Supplementary analyses in subpopulations or using alternative methods demonstrated EFS HR ranging from 0.43 (95% CI: 0.23–0.79) to 0.59 (95% CI: 0.28–1.27) and OS HR ranging from 0.29 (95% CI: 0.11–0.72) to 0.45 (95% CI: 0.21–0.98)^4^. Common adverse reactions (≥5%) in study 3b, including laboratory abnormalities, were otitis media, diarrhea, cough, sinusitis, pneumonia, upper respiratory tract infection, conjunctivitis, vomiting, pyrexia, allergic rhinitis, decreased neutrophils, increased ALT (alanine aminotransferase), increased AST (aspartate aminotransferase), hearing loss, and skin infection^4^.

Future recommendations include continued research to explore eflornithine’s efficacy in various neuroblastoma subtypes and its potential in combination with other therapies. Further studies should be conducted to monitor the long-term effects of eflornithine treatment on patients, assessing both efficacy and potential late side effects. Also identification and validation of biomarkers that can predict patient response to eflornithine is necessary, enabling more precise treatment strategies.

## Ethical approval

Ethics approval was not required for this article.

## Consent

Informed consent was not required for this article.

## Sources of funding

Not applicable.

## Author contribution

All authors were involved in the conceptualization of ideas and critical reviews with comments. All authors approved the final manuscript.

## Conflicts of interest disclosure

The author declares no conflict of interest.

## Research registration unique identifying number (UIN)

Not applicable.

## Guarantor

Abubakar Nazir, Oli Health Magazine Organization, Research and Education, Kigali, Rwanda; E-mail: abu07909@gmail.com; ORCID ID: 0000-0002-6650-6982.

## Data availability statement

Not applicable.

## Provenance and peer review

Not commissioned, externally peer-reviewed.
